# Subcapsular Hepatic Hematoma Secondary to Preeclampsia With a Successful Maternal Outcome: A Case Report

**DOI:** 10.7759/cureus.80911

**Published:** 2025-03-20

**Authors:** Rashid Sadek Azar, Jocelyn M Leon Turriza, Roberto Velazquez Tass, Roxana Guillemin Sosa Montejo

**Affiliations:** 1 Obstetrics and Gynecology, Hospital General de Especialidades, Campeche, MEX

**Keywords:** damage control laparotomy, liver lesion, preeclampsia with hellp syndrome, preeclampsia with severe features, subcapsular hepatic hematoma

## Abstract

Subcapsular hepatic hematoma (SHH) is a rare and life-threatening condition, often leading to severe maternal complications. Its diagnosis is frequently challenging due to nonspecific symptoms and a reliance on ultrasound findings. Case reports for this type of pregnancy complication are limited due to its low incidence.

A 30-year-old pregnant woman at 32.3 weeks with preeclampsia with severe features presented with epigastric pain and high blood pressure, requiring an emergency C-section. She developed a peri-hepatic subcapsular hematoma, leading to hypovolemic shock and multiple surgeries, including liver packing. After stabilization and follow-up, she was discharged without complications.

We reported a rare case of a patient with hepatic subcapsular hematoma secondary to preeclampsia with severe features. SHH is a rare but serious pregnancy complication, often linked to hemolysis, elevated liver enzymes, and low platelets (HELLP) syndrome and preeclampsia, with a high maternal mortality rate. Diagnosis is challenging due to nonspecific symptoms, requiring imaging studies such as ultrasound or CT. Treatment varies from conservative management to surgical intervention, depending on hemodynamic stability.

## Introduction

A subcapsular hepatic hematoma (SHH) is an accumulation of blood located between Glisson's capsule and the liver parenchyma. Its incidence during pregnancy is between 2% and 8%, with a high maternal mortality risk ranging from 17% to 59%, particularly in cases associated with hemolysis, elevated liver enzymes, and low platelets (HELLP) syndrome and preeclampsia with severe features [1]. The clinical presentation of a SHH is nonspecific, making diagnosis challenging. Diagnosis is confirmed through imaging studies, with ultrasound as the first-line option, followed by CT and MRI [2-5]. The treatment of SHHs can be conservative or non-conservative, depending on the patient's hemodynamic stability and the severity of the liver injury [1,2,4].

## Case presentation

A 30-year-old female, currently in her second pregnancy with a previous miscarriage and no other significant medical history, was at 32.3 weeks of gestation with a diagnosis of gestational hypertension, previously managed with methyldopa 500 mg every 8 hours. She presented to the obstetric ED with epigastric pain radiating to the right shoulder for one week, accompanied by elevated blood pressure readings of up to 180/105 mmHg at the time of evaluation. This required the administration of two boluses of hydralazine 5 mg and the initiation of neuroprotection with magnesium sulfate using a modified Zuspan protocol.

Upon admission to the obstetric surgery unit, her blood pressure remained above the target range (160/110 mmHg), accompanied by epigastric pain radiating to the shoulder and other signs of vasospasm. Laboratory tests were ordered, revealing the following results: hemoglobin 13.8 g/dL, hematocrit 39%, platelets 145,900/mm³, aspartate aminotransferase (AST) 83 U/L, alanine aminotransferase (ALT) 111 U/L, and a urinalysis showing protein levels of 30 mg/dL (Table 1). Due to the persistence of symptoms, an urgent cesarean section was performed to improve maternal and fetal outcomes.

**Table 1:** Patient's laboratory results from initial admission to postoperative period after the second surgery. AST: Aspartate aminotransferase; ALT: Alanine Aminotransferase.

Laboratory Parameter	Initial Admission	Postoperative (After First Surgery)	Postoperative (After Second Surgery)	Normal Range
Hemoglobin (g/dL)	13.8	9.52	7.21	12-15
Hematocrit (%)	39	26	21	36-48
Platelets (/mm³)	145,900	157,500	131,900	150-450
AST (U/L)	83	87	500	15-38
ALT (U/L)	111	1000	907	13-40
Proteinuria (mg/dL)	30	Not reported	Not reported	Not detected

A male newborn was delivered at 05:30 AM, weighing 1800 g, with an Apgar score of 3-5, gestational age of 33 weeks, and a length of 45 cm. The infant had no muscle tone and required two cycles of positive pressure ventilation, orotracheal intubation, and admission to the Neonatal Intensive Care Unit (NICU). Additional findings included scant, clear amniotic fluid, a normal umbilical cord, an anterior placenta with 20% abruption, and two fibroids on the anterior uterine wall measuring approximately 1×1 cm. The total blood loss was 800 cc.

**Figure 1:**
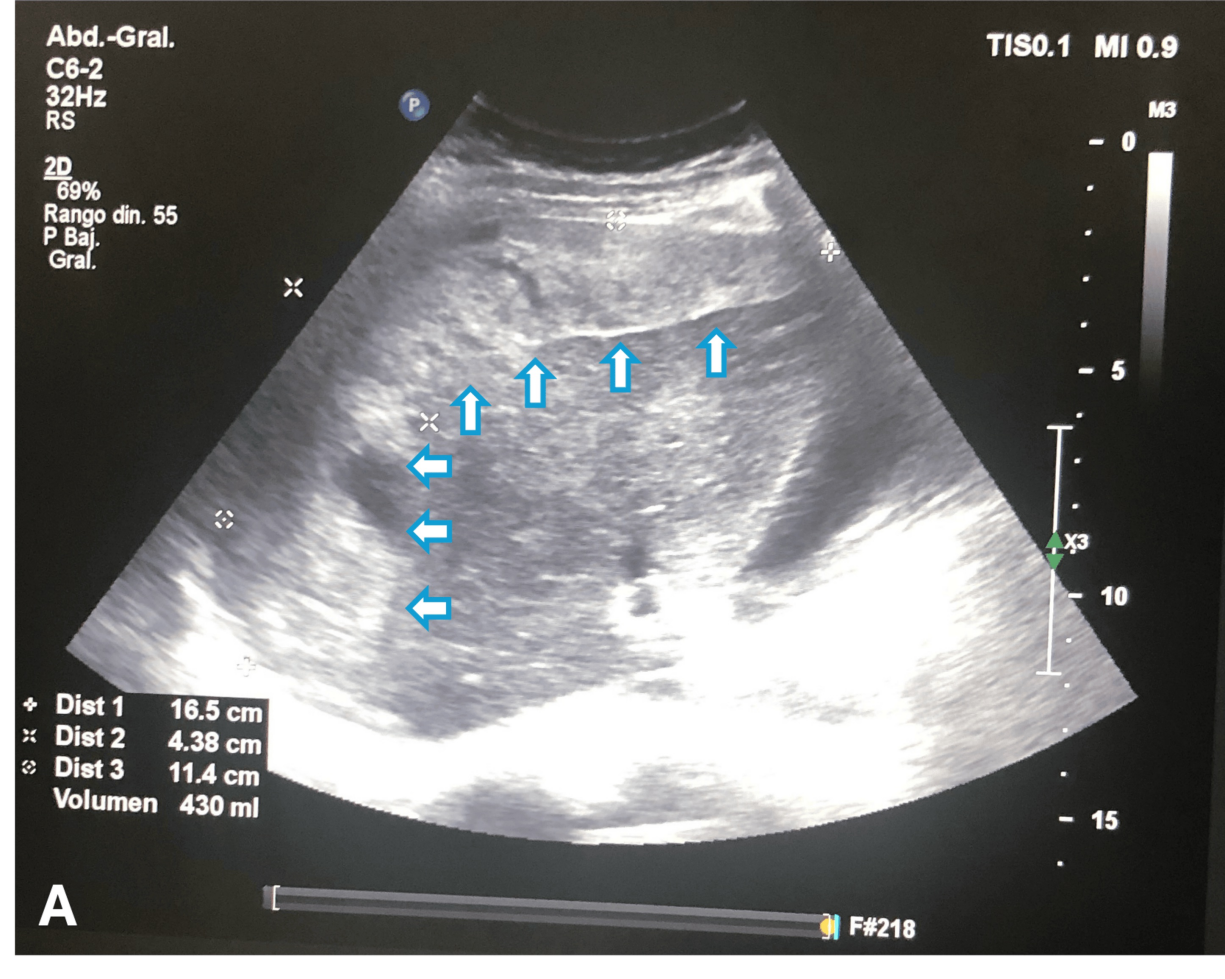
Abdominal ultrasound showing a subcapsular hepatic hematoma in the right lobe.

At the end of the procedure, during monitoring, signs of hypovolemic shock were detected, with a blood pressure of 70/40 mmHg and a heart rate of 130 bpm. A maternal code was activated, and fluid resuscitation along with vasopressor support was initiated. An intraoperative abdominal ultrasound revealed a peri-hepatic subcapsular hematoma predominantly affecting the right lobe, measuring approximately 16.5 × 4.3 × 11.4 cm (Figure 1), with an estimated volume of 430 ml. Additionally, there was non-quantifiable free fluid in the perisplenic region, approximately 60 ml in the left paracolic gutter, 50 ml in the right paracolic gutter, and 80-100 ml in the periuterine pelvic cavity. The uterus was involuting, with post-surgical changes in the myometrium and no evidence of retained placental tissue. Laboratory results showed hemoglobin 9.52 g/dL, hematocrit 26%, platelets 157,500/mm³, AST 87 U/L, and ALT 1000 U/L (Table 1).

**Figure 2:**
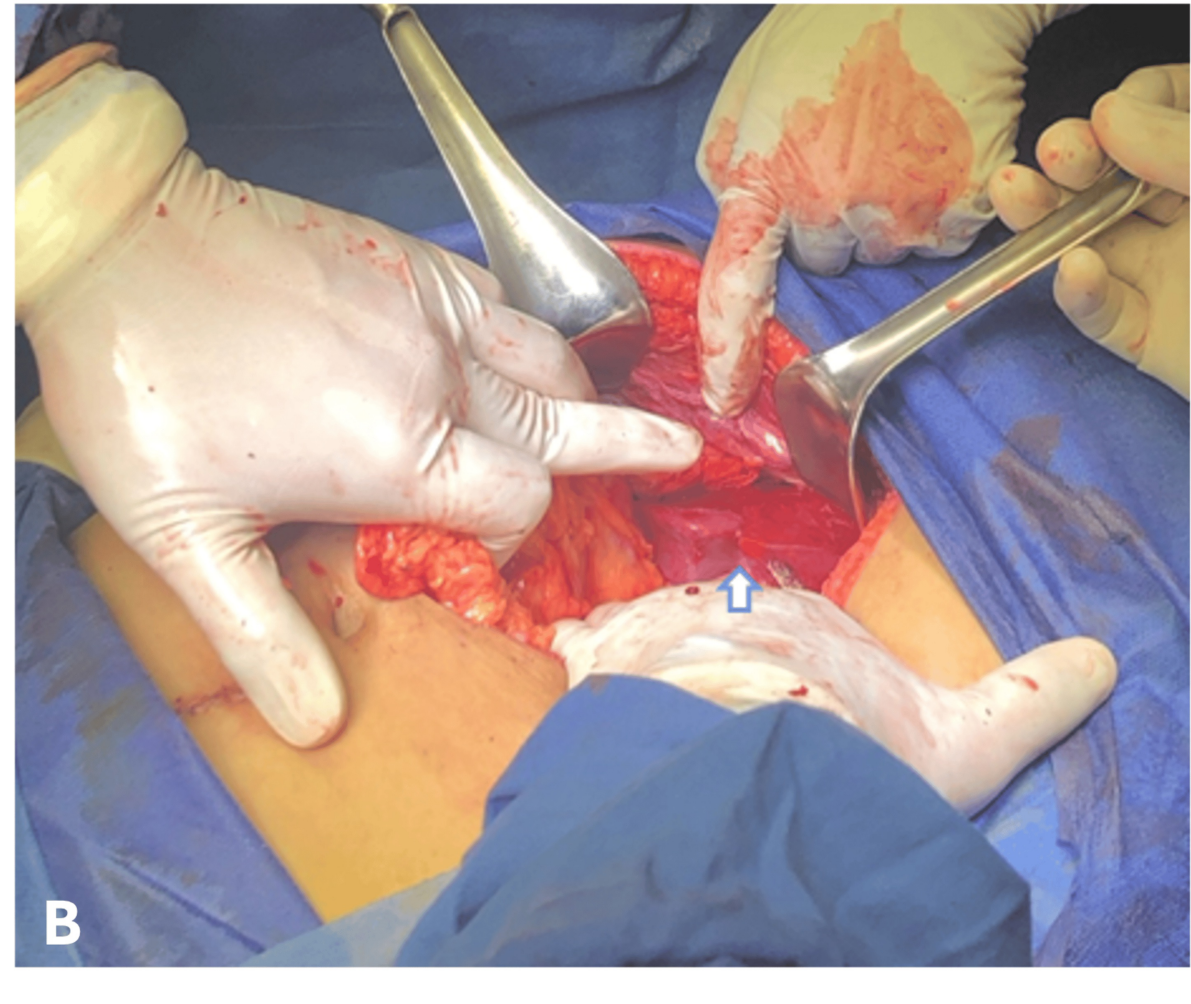
Hepatic laceration is observed following the drainage of a subcapsular hepatic hematoma.

An emergency exploratory laparotomy was performed by the General Surgery team, revealing 800 ml of hemoperitoneum and a ruptured SHH measuring 15 cm in segments VI and VII (Figure 2). The liver was packed with five gauze pads wrapped in a latex glove. A ¾-inch Penrose drain was placed in the foramen of Winslow and exteriorized through a counter-incision in the right flank.

Following surgery, the patient was admitted to the adult ICU. Four hours later, a second maternal code was activated due to active, non-quantifiable bleeding from the Penrose drain. A reoperation was performed by the General Surgery team, revealing an extension of the hematoma toward the posterolateral liver with 850 cc of hemoperitoneum. A second packing procedure was performed using six gauze pads after dissecting the round and cardinal ligaments of the liver, with intact hepatic veins observed.

Forty-eight hours after the second surgery, unpacking and abdominal closure were performed. Six gauze pads were removed from the hepatic bed without active bleeding; however, a hematoma in segments VI and VII with 150 ml of blood was noted. This was managed conservatively by the General Surgery team.

Postoperative laboratory tests showed hemoglobin 7.21 g/dL, hematocrit 21%, platelets 131,900/mm³, AST 500 U/L, and ALT 907 U/L (Table 1). The patient was discharged without complications 48 hours after the last surgical event, with a follow-up scheduled at six weeks, during which no further complications were reported.

## Discussion

Subcapsular hepatic hematoma is a rare but severe complication of hypertensive disorders of pregnancy, particularly preeclampsia with severe features and HELLP syndrome. It occurs due to hepatic vasospasm, endothelial dysfunction, and microvascular injury, leading to hemorrhage beneath the liver capsule [3]. If left undiagnosed or untreated, SHH can rupture, resulting in massive hemoperitoneum, hemorrhagic shock, and high maternal-fetal mortality [2]. In this case, the patient presented with epigastric and right shoulder pain, a hallmark symptom of hepatic involvement in preeclampsia. Despite antihypertensive management with methyldopa, her blood pressure remained severely elevated, necessitating emergency cesarean delivery. The presence of persistent pain and signs of hemorrhagic shock postoperatively prompted urgent imaging, leading to the diagnosis of a ruptured hepatic hematoma. Immediate surgical intervention, including liver packing and damage control resuscitation, was essential for hemodynamic stabilization.

The management depends on its size, stability, and presence of rupture [2,5]. In stable, unruptured cases, conservative management with close monitoring may suffice. However, in cases like this one, where a rupture occurs, surgical intervention is life-saving [2,5]. Treatment options include hepatic packing, embolization, or even partial hepatectomy in severe cases [5]. This case demonstrates the importance of a high index of suspicion in hypertensive pregnant patients with unexplained epigastric pain, as timely intervention can prevent maternal and fetal morbidity and mortality.

Despite a challenging clinical course, the patient had a favorable outcome due to early recognition, multidisciplinary surgical management, intensive postoperative care, and the patient made a full recovery without long-term complications. This case underscores the critical role of prompt diagnosis, aggressive surgical intervention, and vigilant hemodynamic monitoring in managing hepatic complications of severe preeclampsia.

## Conclusions

This case highlights the rare but life-threatening complication of SHH in the context of preeclampsia with severe features. The patient's initial presentation with epigastric pain, right shoulder pain, and severe hypertension was indicative of hepatic involvement, which ultimately led to the rupture of a subcapsular hematoma following delivery. Early recognition and multidisciplinary management, including urgent abdominal delivery, intraoperative ultrasound, exploratory laparotomy, and sequential surgical interventions, were critical in preventing maternal mortality. This case underscores the importance of early diagnosis, close monitoring, and timely surgical intervention in managing hepatic complications of hypertensive disorders in pregnancy.
